# A role for PaxB in regulating blebbing: experimental insights and theoretical perspectives from *Dictyostelium discoideum*

**DOI:** 10.21203/rs.3.rs-8554870/v1

**Published:** 2026-01-19

**Authors:** Zully Santiago, Brian Wey, Sobana Handi Dinuka Sewwandi de Silva, Jessica Reznik, Lamina Siby, Emmanuel Asante-Asamani, Derrick Brazill

**Affiliations:** 1* Mathematics, Clarkson University, 8 Clarkson Avenue, Potsdam, 13699, NY, US.; 2 Academic Affairs, York College, 94-20 Guy R Brewer Blvd, Jamaica, 11451, NY, US.; 3 Natural Sciences, Baruch College, 55 Lexington Ave, New York, 10010, NY, US.; 4 Mathematics, University of Kelaniya, Kelaniya, Sri Lanka.

**Keywords:** PaxB, *Dictyostelium*, Chemotaxis, Blebbing

## Abstract

Eukaryotic cells migrate using pressure-driven blebs or actin polymerization driven pseudopods, with cells preferring to bleb in compressed environments where high protrusion forces are required for movement. In mammals, paxillin is a focal adhesion protein that acts as a scaffold, linking integrins to the actin cytoskeleton and recruiting signaling molecules that regulate adhesion, cytoskeletal remodeling, and migration. *Dictyostelium possesses* a paxillin ortholog, PaxB, which shares conserved domains with mammalian paxillin and participates in processes such as adhesion, cytokinesis, development,and chemotaxis. However, the role of PaxB in blebbing is not well understood. Our work combines experimental and theoretical methods to elucidate the role of PaxB in blebbing. We use an under-agarose assay to collect data on *paxB*^−^ and wild-type cells under low and high compression and observe blebbing characteristics such as area and frequency. Our results point to a role for PaxB in regulating the relative size of blebs in response to increased compression through modulating the quality of the newly reformed cortex. Using a mathematical model, we show that decreasing the assembly rate of the cortex during bleb formation leads to relatively larger blebs, thus supporting our experimental findings. Together, our experiments and theory suggest a new role of PaxB in bleb-based chemotaxis.

## 1 Introduction

Chemotaxis, the directed movement of cells in response to chemical gradients, is fundamental to numerous biological processes, including embryonic development, immune responses, and tissue organization[1]. Without chemotaxis, the complex cellular rearrangements required for organismal development would not occur. Historically, much of our understanding of chemotaxis and actin-based migration comes from studies in two-dimensional (2D) environments, where cells migrate across flat surfaces. While these studies have provided valuable insights, they do not fully capture the conditions cells encounter in vivo, where migration occurs in three-dimensional (3D) environments characterized by compression, shear stress, and confinement [2, 3]. These differences suggest that the molecular mechanisms governing chemotaxis may vary significantly between 2D and 3D contexts.

Two major modes of motility have been described: actin-based migration, which relies on structures such as lamellipodia, filopodia, and pseudopodia[4], and bleb-based migration, which predominates in 3D environments[5]. In bleb-based motility, the plasma membrane locally detaches from the actin cortex, forming blister-like protrusions that drive forward movement [6]. This mechanism is utilized by diverse cell types, including cancer cells, immune cells, and the social amoeba *Dictyostelium discoideum* (*D. discoideum*)[7-10]. Despite its biological importance, the molecular and biophysical principles underlying bleb initiation and coordination remain poorly understood.

Recent efforts have emphasized the value of mathematical modeling in describing cell shape and motility[10-12]. Because blebs dominate migration in 3D environments, representing their geometry and mechanics mathematically has provided critical insights into how physical constraints and cellular architecture influence chemotactic behavior. Consequently, a combination of experimental approaches and computational modeling has provided insight into the role of different proteins in bleb-based chemotaxis. These studies describe the role of actin and myosin dynamics in bleb nucleation and stabilization[13]. Additionally, they suggest that bleb size is regulated by membrane stiffness, cortical tension, and intracellular pressure, with TalA, a talin homolog in *Dictyostelium*, being shown to have a role in bleb-based chemotaxis through influencing bleb number and size, most likely by modulating intracellular pressure [14, 15].

Talin and paxillin are known to bind to each other at focal adhesions to stabilize adhesion complexes and coordinate cytoskeletal remodeling[16, 17]. This interaction integrates mechanical and biochemical signaling, influencing adhesion strength and migration dynamics. In mammals, paxillin is a focal adhesion protein that acts as a scaffold, linking integrins to the actin cytoskeleton and recruiting signaling molecules that regulate adhesion, cytoskeletal remodeling, and migration[18]. *Dictyostelium* possesses a paxillin ortholog, PaxB, which shares conserved domains with mammalian paxillin and participates in processes such as adhesion, cytokinesis, development, and chemotaxis[19, 20]. However, whether paxillin contributes to bleb-based motility remains unclear. Intriguingly, studies in mammalian systems suggest that paxillin loss enhances invasion in 3D environments, raising the possibility that paxillin may influence bleb formation or dynamics[21]. Understanding this relationship could reveal new principles of cell migration in confined environments.

Here, we investigate the role of PaxB in bleb-based chemotaxis in *Dictyostelium* using experimental and modeling approaches. We find that PaxB regulates the relative size of blebs, much like TalA, and this regulation is most likely mediated through modulation of actin polymerization.

## 2 Methods

### Cell culture

*Dictyostelium discoideum* cells, Ax2 (wild-type), paxB^−^, Ax2-LifeAct-GFP, and paxB^−^ LifeAct-GFP, were grown axenically in HL5 nutrient medium with glucose (ForMedium), supplemented with 100 IU/mL penicillin and 100*μg/mL* streptomycin (Amresco). Cells were grown in shaking culture at 150 rpm at 22°*C*. Additionally, nutrient media for paxB^−^ cells was supplemented with 10 *μg/ml* Blasticidin [19]. All Life-Act expressing lines were supplemented with G418 4-20 *μg/mL* [12]. All cell lines were in log phase (1 × 10^6^ cells/mL to 4 × 10^6^ cells/mL) from shaking prior to use for experiments.

### LifeAct-GFP expression in cells

To create LifeAct-GFP expressing lines, Ax2 and *paxB*^−^ cells were transformed in the same manner as described in [12] with Life-Act-GFP plasmid. To confirm lines expressing LifeAct-GFP, isolates were suspended in PBM in an 8-well chambered 1 German borosilicate sterile coverglass slide (ThermoFisher). Clones were chosen for imaging based on similarity to F-actin LifeAct-GFP fluorescense labeling found in previous work in *D. discoideum* [23].

### Cyclic-AMP (cAMP) under agarose assay

Blebbing activity of chemotaxing Ax2 and *paxB*^−^ LifeAct expressing cells was visualized using a cAMP (cyclic AMP) under-agarose assay. A number 1 German borosilicate sterile 2-well chambered cover glass slide (ThermoFisher) was preheated (90°C, 1 *min*) and loaded with 750*μL* of melted 0.4% or 0.7% Omnipur agarose (EMD Millipore) laced with 1mg/mL of 70,000 MW Rhodamine B isothiocyanate-dextran (Sigma-Aldrich). After solidification of the agarose gel, we created two wells, one containing 4*μM* cAMP to establish a gradient and the other containing 1 × 10^5^ to 2 × 10^5^ cAMP competent cells. Details on the construction of the well and time duration for setting up the gradient can be found in [12].

### Live imaging and microscopy

Chemotaxing cells were imaged using a Leica DMI-4000B inverted microscope (Leica Microsystems Inc.) mounted on a TMC isolation platform (Technical Manufacturing Corporation) with a Yokogawa CSU 10 spinning disc head and Hamamatsu C9100-13 ImagEM EMCCD camera (Perkin Elmer) fitted with diode lasers of 491 nm, 561nm, and 638 nm (Spectra Services Inc.) [24]. The 491 nm laser was used to excite LifeAct-GFP whereas the 561nm laser excited RITC-dextran. As cells crawled under a RITC-dextran laced agarose gel, the cortex was visualized by LifeAct-GFP while membrane position was visualized as a negative against the RITC background. Cells were imaged for a duration of 30 seconds using a 100x/1.44 oil immersion objective at maximum camera speed. Exposure time for GFP was 0.800 seconds and 0.122 seconds for the RITC channel. Consequently we achieved a frame rate of ~ 1.7 – ~ 1.9 seconds/frame. We utilized the image acquisition software Volocity 5.3.3 (Perkin-Elmer) and processed the final images with the open source software Image J (https://imagej.nih.gov/ij/).

### Image processing

The raw data for each series, consisting of the membrane and cortex channels, were split and separated from each other and adjusted for optimal brightness and contrast using ImageJ. Once complete, the two channels (membrane and cortex) were merged together with the cortex in green and the membrane in red. The merged images were subsequently saved as an .avi file without any compression to be used for detection of blebs and calculation of bleb area.

### Determining bleb frequency, bleb area and cell area

We analyzed the first 30 seconds of live imaging to determine the bleb frequency and area. To detect blebs, we looked for a clear separation of the membrane (from RITC-dextran channel) and a reformation of the cortex (from LifeAct-GFP channel). The number of blebs generated by a cell within the observation period was recorded as the frequency. The bleb area was calculated from the first frame for which a stable bleb size was observed. We considered a bleb to have obtained a stable size if subsequent frames did not show evidence of further enlargement of the bleb. This was evidenced by either a fully detached membrane (from the RITC-dextran channel) or clearly outlined cortex (in the LifeAct-GFP channel) or both. In either case, we confirmed that the structure being observed was a bleb by looking for evidence of the three phases of bleb formation, membrane separation, cortex reformation, cortex degradation [12], using the plot profile feature in Image J. The polygonal tool in ImageJ was used to create an outline of the bleb. The resulting boundary was smoothed using the spline fit tool. Bleb area measurements were obtained from the Analyze menu in ImageJ.

Cell area was calculated using QUIMP plugin in ImageJ. The LifeAct-GFP live imaging frames of each cell were made into a stack in Image J and processed using the University of Warwick’s Quimp add-on. QuimP converted the stack into 8-bit images, and ImageJ’s selection tool was used to encircle the cell of interest in each video. Each frame was reviewed to ensure the add-on accurately encircled the perimeter of the cell prior to saving. The resulting QuimP data provided the following: centroid positioin, coordinates, displacement, distance traveled, directionality, speed, perimeter,elongation, circularity, and cell area.

### Statistical analysis

Bleb and cell area as well as bleb frequency measurements were obtained from multiple experiments under high and low compression condition. Bar graphs were created to visualize the distribution of the bleb frequency and area. The student’s t-test was used to check for significant differences between groups. Statistical analysis was done in R.

### Theoretical model of bleb expansion

#### Geometric assumptions

**Fig. 1:**
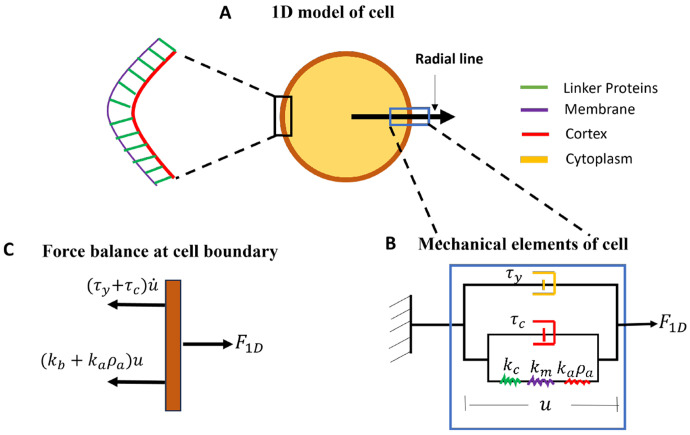
Geometric and mechanical assumptions for mathematical model. A) 1D mechanical element of the cell. B) 2D organization of 1D mechanical elements. C) Illustration of bleb initiation process. Lighter color denotes lower pressure, P.

Bleb-based migration occurs in three-dimensional space with cells often modeled as spheres and blebs as hemispherical protrusions. In our experiments, cells crawl on the surface of a two-dimensional slide under the weight of an agarose gel which creates a pseudo three-dimensional environment. Under such confinement, cells adopt a ‘pancake’ shape with an almost uniform vertical cross section. In this context, the cell geometry can be simplified to two-dimensions by considering change in the cell shape during blebbing on a fixed horizontal plane. To further simplify the geometry and complexity of the model, we will track bleb expansion by measuring the displacement of the detached membrane from the old cortex along a radial line drawn from the center of the cell pointing outwards. See Fig. 1A for an illustration of the radial line.

#### Mechanical assumptions

During blebbing, the cell boundary extends due to motion of cytoplasmic fluid. Our primary assumption about the cytoplasm is that it is a viscous fluid with viscosity coefficient $\tau_{y}\;(nNs∕\mu m^{3})$. This assumption has been used in other bleb expansion models and found to yield biologically relevant bleb dynamics [25]. We consider the cell boundary as a composite body comprising of an outer membrane which is supported by an inner cortex with linker proteins connecting the two structures. The membrane is a phospholipid bilayer, modeled as an elastic material with elastic stiffness $k_{m}\;(nN∕\mu m^{3})$. The dense network of crosslinking and motor proteins holding branched actin filaments together in the cortex give it elastic and viscous properties, with the viscous behavior stemming from the dynamic renewal of actin filaments through polymerization and depolymerization [26, 27]. Thus, the cortex can be considered as a Kelvin-Voigt viscoelastic solid, with viscosity coefficient $\tau_{c}\;(nNs∕\mu m^{3})$ and elastic stiffness $k_{c}\;(nN∕\mu m^{3})$. Proteins linking the membrane to the cortex undergo continuous binding and unbinding, with a constant binding rate $k_{on}\;(s^{-1})$ and a force dependent unbinding rate $k_{off}\;(s^{-1})$. At any point along the boundary, the local density of bound linkers, $\rho_{a}\;(\mu m^{-2})$ is assumed to induce an elastic resistance of $k_{a}\rho_{a}$ to membrane separation with stiffness $k_{a}\;(nN∕\mu m)$ per linker protein.

Since the motion of the cell boundary is driven by the flow of the cytoplasm, we assume that these two components (cytoplasm and boundary) experience the same velocity $\dot{u}\;(\mu m∕s)$ with displacement u(μm). Note that the displacement u is a scalar quantity in 1D since it is measured along a radial line through the cell boundary, as shown in Fig. 1A. The assumption of a common velocity for cytoplasm and boundary represents a no-slip condition which has been used in recent models of bleb formation [25]. We represent the common displacement of the cytoplasm and boundary by arranging their mechanical elements in parallel, as shown in Fig. 1B.

Prior to bleb initiation, the linker proteins inhibit separation of the membrane from the cortex. The separation of the membrane from the cortex creates a purely elastic boundary which expands under the force of fluid pressure. Shortly after membrane detachment, a new cortex begins to form under the protruded membrane giving the bleb boundary viscoelastic properties. These observations motivate our treatment of the boundary as a single viscoelastic structure with time-dependent mechanical properties. Its elastic component has stiffness contributions from the membrane $\left(k_{m}\right)$, cortex $\left(k_{c}\right)$ and linker proteins $\left(k_{a}\rho_{a}\right)$, arranged in series as shown in Fig. 1B. The viscous component, purely due to the cortex viscosity $\tau_{c}$, is arranged in parallel with the elastic element. As a new cortex is formed beneath the protruded membrane, the old cortex is degraded. The degradation alters the porosity of the cortex and viscosity of the cytoplasm within the bleb, potentially regulating the entry of large proteins, organelles and macromolecules into the bleb. Thus, mechanically the cytoplasm within the bleb (bleb cytoplasm) acts as a damper with time dependent damping coefficient $\tau_{y}(t)$.

#### Submodel for boundary velocity, adhesion density and applied force

Let $F_{1D}\;(nN∕\mu m^{2})$ denote the force density from intracellular fluid pressure that drives the expansion of blebs. Then, by our assumption of a parallel arrangement of the cytoplasm and boundary, the force driving bleb expansion is distributed across the cytoplasm $\left(F_{1D}^{cyto}\right)$ and cell boundary $\left(F_{1D}^{bnd}\right)$ and satisfies the force balance equation

(1) $$F_{1D}=F_{1D}^{cyto}+F_{1D}^{bnd}$$

at any point on the cell boundary. The force contribution from the elastic component of the boundary is $\left(k_{b}(t)+k_{a}\rho_{a}\right)u$, where $k_{b}(t)$ is the time-dependent contribution to boundary stiffness from the cortex and membrane (see illustration in Fig. 1C). The force from the viscous component of the boundary is proportional to its deformation rate and can be expressed as $\tau_{c}(t)\dot{u}$. Thus, we can write the force across the cell boundary as $F_{1D}^{bnd}=\left(k_{b}(t)+k_{a}\rho_{a}\right)u+\tau_{c}(t)\dot{u}$. The resistance of the bleb cytoplasm to deformation is proportional to the deformation rate $\dot{u}$ with the force across it modeled as $F_{1D}^{\text{cyto }}=\tau_{y}(t)\dot{u}$ (see illustration in Fig. 1C). Combining our forces across the cytoplasm and boundary as shown in Eq. 1 we obtain the force balance

(2) $$F_{1D}=\left(k_{b}(t)+k_{a}\rho_{a}\right)u+\left(\tau_{y}(t)+\tau_{c}(t)\right)\dot{u}$$

which can be solved for the boundary velocity as

(3) $$\frac{du}{dt}=\frac{F_{1D}}{\left(\tau_{c}(t)+\tau_{y}(t)\right)}-\frac{\left(k_{b}(t)+k_{a}\rho_{a}\right)}{\left(\tau_{c}(t)+\tau_{y}(t)\right)}u$$

The dynamics of the density of linker proteins, $\rho_{a}(t)$ is modeled using the classic adhesion model

(4) $$\frac{d\rho_{a}}{dt}=k_{on}\left(\rho_{0}-\rho_{a}\right)-k_{off}(u(t))\rho_{a}$$

used in Alert’s work in [28] to model membrane peeling during blebbing. Here linker proteins are assumed to bind to the cortex at a rate proportional to the density of unbound linkers $\rho_{0}-\rho_{a}$ with kinetic constant $k_{on}$ and unbind from the cortex at a rate dependent on the boundary displacement u, i.e. $k_{oof}(u)=k_{oof}^{0}e^{\beta k_{a}u\delta }$. Here, $k_{oof}^{0}$ is the rate at which linker proteins detach at zero displacement, β is the thermal energy and δ is the characteristic bond length.

The applied force during bleb expansion $F_{1D}$ is a pressure gradient force, determined by the difference in fluid pressure between the cytoplasm (high pressure) and the small region of detached membrane at nucleation (low pressure) [29]. As the bleb expands, the fluid pressure in the blebbing region increases. The bleb continues to expand until the net pressure between the two regions is zero. In 1D, we we assume this force is initially constant and only varies in time as the boundary expands. We model it as an exponentially decreasing force

(5) $$F_{1D}(u)=F_{0}e^{-mu}(5)$$

with decay rate m and initial value $F_{0}$. This assumption of a constant resting pressure has been used in a recent cell mechanics model [30] to describe process like growth, mitosis and motility. In what follows, we describe the formulation of our time-dependent mechanical parameters, $k_{b}(t),\tau_{c}(t)$ and $\tau_{y}(t)$.

### Submodel for time dependent mechanical properties

In the local region where blebs form, the stiffness and viscosity of the boundary as well as the viscosity of the cytoplasm are very dynamic, as discussed previously. Initially, the separation of the membrane from the cortex creates a purely elastic boundary which expands under the force of pressurized fluid. As a new cortex reforms beneath the expanded membrane, the old cortex (actin scar) is simultaneously degraded. This alters the mechanical properties of the local bleb environment in two ways. First, the stiffness and viscosity of the boundary increases in proportion to the concentration of f-actin that has accumulated beneath the protruded membrane. Secondly, the degradation of the actin scar permits lager proteins, organelles and macromolecules to enter the cytoplasm between the protruded membrane and actin scar (bleb cytoplasm). This tends to increase the viscosity of the bleb cytoplasm in proportion to the concentration of f-actin remaining in the actin scar. Let $k_{b}$ be the boundary stiffness due to the membrane and cortex, then we can model it as

(6) $$k_{b}(t)=k_{m}+r_{1}(t)k_{c}$$

where $0\leq r_{1}(t)\leq 1$ accounts for the proportion of cortex that has reformed beneath the protruded membrane. Likewise, the viscosity of the bleb boundary can be modeled as

(7) $$\tau_{c}(t)=\tau_{c}^{0}r_{1}(t)$$

where $\tau_{c}^{0}$ is the viscosity coefficients of the pre-bleb cortex. When the membrane first detaches from the cortex, the fluid in the bleb cytoplasm is less viscous due to the absence of larger proteins and organelles that could not pass through the cortex. Let $\tau_{y}^{0}$ be the cytoplasmic viscosity prior to bleb expansion, then the initial viscosity of the bleb cytoplasm will be $\theta \tau_{y}^{0}$ where 0<θ<1. We expect the cytoplasmic viscosity to increase as more f-actin is removed from actin scar. Thus, we model the bleb cytoplasmic viscosity as

(8) $$\tau_{y}(t)=\theta \tau_{y}^{0}+(1-\theta )\tau_{y}^{0}r_{2}(t)$$

where $r_{2}(t)$ is the proportion of degraded actin. The precise form of the ratio $r_{1}(t)$ and $r_{2}(t)$ are determined using our recent data-driven modeling of cortex reformation [31]. We set $r_{1}(t)=\frac{k_{ab}^{on}\left(1-e^{-k_{ab}^{oof}t}t\right)}{a_{0}k_{ab}^{oof}}$ as the relative concentration of actin in the reformed cortex. Here, $a_{0}$ is the actin concentration in the mature cortex, $k_{ab}^{on}$ and $k_{ab}^{off}$ are the polymerization and depolymerization rate of actin in the reforming cortex. Similarly, $r_{2}(t)=\left(1-e^{-k_{as}^{oof}t}\right)$ is the relative concentration of actin removed from the degrading cortex, with $k_{as}^{off}$ as the depolymerization rate of actin in the degrading cortex. Details on the derivation of $r_{1}(t),r_{2}(t)$ are provided in the supplemental methods.

Our final model for the time dependent mechanical parameters for the cytoplasmic viscosity, boundary viscosity and boundary stiffness (due to membrane and cortex) thus become

(9) $$\tau_{y}(t)=\theta \tau_{y}^{0}+(1-\theta )\tau_{y}^{0}\left(1-e^{-k_{as}^{off}t}\right)$$

(10) $$\tau_{c}(t)=\tau_{c}^{0}\frac{k_{ab}^{on}\left(1-e^{-k_{ab}^{off}t}\right)}{a_{0}k_{ab}^{off}}$$

(11) $$k_{b}(t)=k_{m}+k_{c}\frac{k_{ab}^{on}\left(1-e^{-k_{ab}^{off}t}\right)}{a_{0}k_{ab}^{off}}.$$

Taken together, Eqns. 3, 4,9, 10, 11, describe the 1D dynamics of bleb expansion. We note that whereas our model for radial displacement of the cell boundary in Eq. 3 is similar to the model for boundary displacement during micropipette aspiration in [32], it is distinct because it accounts for the resistance of linker proteins through the term $k_{a}\rho_{a}$ and couples the viscous element of the cytoplasm in parallel with the viscoelastic element of the cell boundary. The combination of Eq. 3 and Eq. 4 to describe 1D bleb expansion is also unique to this work. Additionally, we have included time-dependent mechanical parameters (Eq. 9-Eq. 11) to capture dynamic changes in the structure of the cell boundary as a new cortex reforms and the old cortex is degraded. Additionally, whereas traditional models of bleb formation compute stretching forces between the cortex and membrane in order to detach linker proteins, our framework uses the displacement of the bleb boundary ((u)) from its original location to compute the unbinding rate of linker proteins and hence the density of linker proteins $\rho_{a}$ available to resist further detachment with stiffness $\rho_{a}k_{a}$.

### Numerical simulation of bleb expansion

We solve the 1D model using MATLAB’s ode23s solver. Given an applied pressure $F_{0}$, we simulate bleb expansion by first determining the boundary displacement and linker density at which the cell boundary and cytoplasmic resistance would balance out the driving pressure. We refer to these as the critical displacement and critical density, denoted by $u^{0}$ and $\rho_{a}^{0}$ respectively. These critical values are necessary to account for the cell’s initial attempt to re-equilibrate forces when the intracellular pressure is increased above resting levels. Note that when this initial displacement is insufficient to balance out the excess pressure, linker proteins are detached in a local region, the membrane separates from the cortex and a bleb is born [33]. Thus, we initiate blebs by setting these critical values as the initial condition for our ode model (Eq. 3 - Eq. 4) and then mimic the detachment of linker proteins by setting $\rho_{a}=0$. The critical displacement and density are obtained by solving for the steady-states of Eq. 3 and Eq. 4 with constant mechanical parameters, since cortex reformation and degradation would not have begun at this time. Consequently, we obtain the following nonlinear solution

(12) $$u^{0}=\frac{e^{-mu^{0}}}{\left(k_{b}+k_{a}\rho_{a}^{0}\right)}\;\rho_{a}^{0}=\frac{k_{on}\rho_{0}}{k_{on}+k_{off}^{0}e^{\delta \beta k_{a}u^{0}}}.$$

which can be solved using MATLAB’s inbuilt nonlinear equation solver. Details on how the steady-state values of the model were calculated has been provided in the supplemental methods. This idea of computing a critical displacement and linker density prior to membrane separation is new to this work and different from existing approaches for initiating blebs [34-37].

## 3 Results

**Fig. 2:**
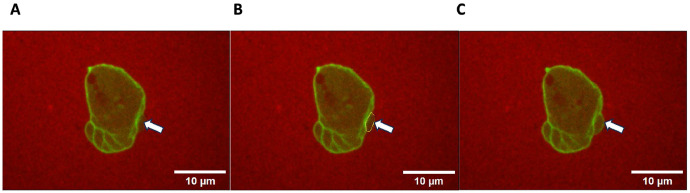
Blebbing *paxB*^−^ cells under 0.7 agarose. A) Cell with membrane separated from cortex, b) region of bleb measured, c) bleb with fully reformed cortex

To investigate the role of PaxB in chemotactic blebbing, wild-type (Ax2) and *paxB*^−^ cells were observed under low (0.4% agarose) and high (0.7% agarose) compression over a 30 second duration. An example of blebbing *paxB*^−^ cell is shown in Fig. 2. Observe that bleb undergoes the complete cycle of membrane separation (Fig. 2A) and reformation of a new cortex beneath the expanded membrane (Fig. 2C). Table. 1 shows the total number of cells and blebs analyzed under each compression condition. In the case of *paxB*^−^, the data was pooled from two independent trials.

### 3.1 Effect of PaxB on the frequency of blebbing

**Fig. 3:**
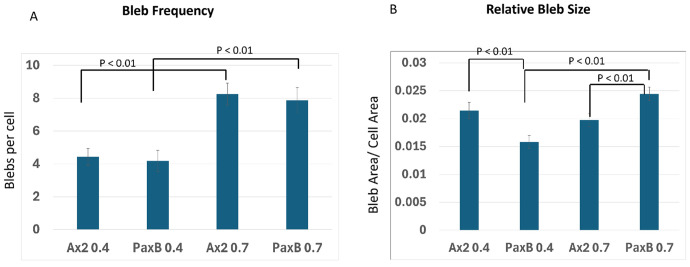
Experimental data. Data on a) frequency of blebs per cell b) relative bleb size

To investigate the effect of PaxB on the frequency of blebbing, we analyzed the number of blebs produced by cells which formed at least one bleb within the 30 second observation window. Results are shown in Fig. 3A. Under low compression, Ax2 and *paxB*^−^ cells produced roughly the same number of blebs, averaging 4.4 ± 0.52 and 4.2. ± 0.64 blebs per cell respectively. Both cells increased blebbing under high compression to 8.25 ± 0.67 for Ax2 and 7.89 ± 0.77 for *paxB*^−^ cells. Estimates are reported as Mean ± SEM. Under both compression conditions there was no significant difference between the wild type and *paxB*^−^ cells. Additionally, in both cell types there was a significant increase in bleb frequency when the cells were exposed to higher compressive force. These data suggest that PaxB does not influence the number of blebs per cell produced by blebbing cells nor the change in their bleb frequency in response to compression.

### 3.2 PaxB regulates the relative size of blebs in response to pressure

Next, we sought to investigate whether PaxB had any effect on the relaive size of blebs produced by cells under different compressive environments. The size of blebs tend to be proportional to the volume of cells, with blebs typically accounting for about 3% of the cell volume. *D. discoideum* cells tend to flatten under the weight of the gel so that they obtain a fairly uniform cross sectional area. Thus, we used the cross sectional area of cells, measured from the focal plane of our confocal microscope, as a measure of cell size. The cross sectional area was quantified using QUIMP plugin in Image J.

To measure the relative size of blebs generated by both cell types, we scaled the bleb area by the cell cross sectional area to obtain a quantity we refer to as the bleb-to-cell area ratio. Observe from Fig. 3B that whereas the bleb-to-cell area ratio of Ax2 is relatively unchanged from 0.0215 ± 0.0015 under low compression to 0.0197 ± 0.0011 under high compression, *paxB*^−^ cells increase their bleb-to-cell area ratio from 0.0158 ± 0.001 to 0.0244 ± 0.0012. Thus, under low compression, the relative size of blebs produced by *paxB*^−^ is smaller than Ax2 but becomes larger than Ax2 under high compression. This is interesting as it suggests that PaxB may regulate the relative size of blebs in response to increased compression.

Work by Pribic et. al [39] found that PaxB, along with phospholipase D regulates actin based processes in *D. discoideum* such as actin polymerization. A major part of the bleb formation cycle is the assembly of a new cortex beneath the detached membrane, which relies on proper actin regulation. In addition to timely localization of actin to the detached membrane, filamentous actin must be properly assembled into a branched network to give the cortex its normal viscoelastic properties. Since paxillin has been found to regulate actin based processes, it stands to reason that a loss of paxillin could influence the timing of actin localization and/or its proper assembly during cortex reformation. It is however, unclear whether altering the assembly rate of the cortex would have any effect on the size of blebs that form. In the remainder of the work, we investigate the latter question using mathematical modeling. Results from our theoretical investigation will help in formulating a hypothesis on a potential mechanism by which PaxB may regulate relative bleb size in response to increased compression.

### 3.3 A mathematical model of bleb expansion with actin dynamics

We have developed a one-dimensional mathematical model that describes bleb expansion by quantifying the displacement u of the cell membrane away from the old cortex along a radial line drawn from the center of the cell outwards (see Fig. 1A for an illustration). An important component of the model is its ability to account for the dynamics of actin during the reformation of a new cortex beneath the protruded membrane as well as the degradation of the old cortex. The inclusion of cortex reformation dynamics is necessary for us to investigate how regulating actin assembly rate can affect bleb size on a realistic bleb expansion time scale. Details of the model development with its simplifying assumptions for the geometry and mechanics of the cell has been described in the Methods section. We provide a brief, more intuitive summary here.

The primary equation in the model tracks the boundary velocity of the bleb $\frac{du}{dt}$ and is given by Eq. 3, repeated here for continuity of the discussion

$$\frac{du}{dt}=\frac{F_{1D}}{\left(\tau_{c}(t)+\tau_{y}(t)\right)}-\frac{\left(k_{b}(t)+k_{a}\rho_{a}\right)}{\left(\tau_{c}(t)+\tau_{y}(t)\right)}u.$$

The boundary velocity, increases in proportion to the driving force for bleb expansion $F_{1D}$ and is resisted by the viscosities of the cytoplasm $\tau_{c}(t)$ and cortex $\tau_{y}(t)$. The expression for the driving force is given in Eq. 5. The cytoplasmic and cortex viscosities are defined as functions of time because they change as the bleb expands. Specifically, $\tau_{c}(t)$ is the cytoplasmic viscosity within the bleb which gradually increases in value as larger proteins and organelles enter the bleb when the old cortex degrades. The cortex viscosity $\tau_{y}(t)$ also increase as the cortex reforms beneath the detached membrane. The precise models for these mechanical parameters are given in Eq. 9 and Eq. 10. The second term on the right hand side of the boundary velocity equation accounts for the resistance to bleb expansion due to the elastic stiffness of the cortex and membrane $k_{b}(t)$ as well as the linker proteins that bind the membrane to the cortex $k_{a}\rho_{a}(t)$. The model for the elastic stiffness $k_{b}(t)$ is given in Eq. 11. The density of linker proteins $\rho_{a}(t)$ is also time-dependent due to the dynamic binding and unbinding of these proteins. We model the dynamics of these linker proteins using an existing model developed by [28] presented in Eq. 4. Details of how we simulate bleb expansion is also given the Methods section. To compare our results with the experimental data we compute the relative bleb size by scaling the displacement of the bleb to the diameter of a typical *Dictyostelium* cell, which is about 10μm.

First, we validate our mathematical model by exploring its ability to replicate known effects of cell mechanical parameters on bleb size and then apply it to test our stated hypothesis. Recent experimental data on the dynamics of bleb formation in *Dictyostelium discoideum* cells suggests that blebs reach maximum size within the first 5-10 seconds after membrane separation and stabilize by 25 seconds [31]. Therefore, we simulate our model for 10-25 seconds. All model parameters used in the simulation are presented in Table 2, with their corresponding units and references.

### 3.4 Effect of cell viscoelastic properties on bleb size

When the membrane first detaches from the cortex it expands under the force of flowing cytosol, driven by intracellular pressure and resisted only by its stiffness, $k_{m}$ and initial fluid viscosity $\tau_{y}$. As the cortex begins to reform beneath the membrane and degrade at the old location of the cortex, the boundary resistance increases and slows down bleb expansion. It is well known that bleb size increases with increasing intracellular pressure and decreases as the viscoelastic properties of the cortex and membrane increases [5, 6, 25]. It has also being observed experimentally that as actin reforms beneath the protruded membrane, the bleb boundary undergoes a partial retraction as it approaches steady-state size [31]. We sought to test our model’s ability to capture these known trends by varying our model parameters. We allowed the cortex-free bleb to expand for 2 seconds before beginning cortex reformation, as was determined from experimental data in our previous study [31].

**Fig. 4:**
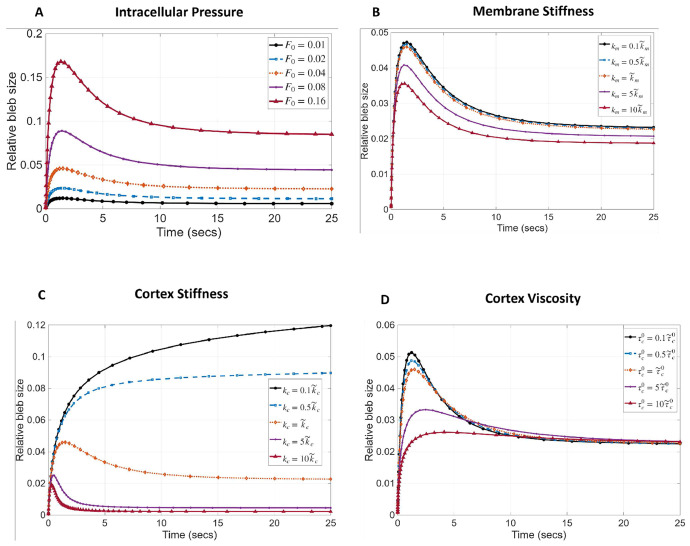
Influence of cell mechanical properties on bleb size. Effect of A) Intracellular pressure B) membrane stiffness, C) cortex stiffness and D) cortex viscosity. Bleb size is measured as the strain of the cell boundary at a single point. $\tilde{r}$ is the baseline value of parameter r reported in Table 2.

Our results in Fig. 4 demonstrate the expected dynamics of a bleb. That is, a rapid expansion within the first 2 seconds and slower expansion or retraction to an equilibrium size after cortex reformation is initiated. We varied the resting intracellular pressure $F_{0}$ and membrane stiffness $k_{m}$, cortex stiffness $k_{c}$ and baseline cortex viscosity $\tau_{c}^{0}$ by scaling their baseline values given in Table 2. Observe from Fig. 4A that bleb size increases with intracellular pressure. Similarly, as membrane elastic stiffness decreases, the bleb expands faster to a higher maximum size and then retracts to a larger equilibrium size (Fig. 4B). As the cortex stiffness decreases, blebs expand faster and settle down to a larger equilibrium size (Fig. 4C). Interestingly, there appears to be two separate bleb expansion regimes regulated by cortical stiffness, one in which blebs partially retract to equilibrium size, after reaching their maximum size, and the other where blebs do not retract, but expand gradually to their equilibrium size. Surprisingly, varying cortex viscosity, does not alter equilibrium bleb size, rather it appears to regulate the speed of expansion with a less viscous cortex leading to a larger maximal bleb size (Fig. 4D). Together, these results support the known behavior of expanding blebs and serve as a semi-quantitative validation of our model.

### 3.5 Regulation of actin reformation rate influences relative bleb size

We found it interesting that the equilibrium bleb size did not depend on the cortex viscosity but rather on membrane and cortex stiffness as well as intracellular pressure. Curious about whether this is supported theoretically and also to identify other model parameters influencing the equilibrium bleb size, we analyzed the equilibrium solutions of the model (Eq. 3) with $\rho_{a}=0$ and time dependent parameters described in Eq. 9-Eq. 11. Note that by setting $\rho_{a}=0$, we remove linker proteins from equilibrium analysis since they are detached when the membrane separates from the cortex. In order to perform equilibrium analysis, we converted the 1D model (Eq. 3), with $\rho_{a}=0$, to an autonomous system of differential equation by writing separate rate equations for all time dependent parameters described in Eq. 9-Eq. 11. The resulting steady-state bleb size $u^{*}$ satisfies the condition

(13) $$F_{0}e^{-mu^{*}}-\frac{k_{c}k_{ab}^{on}}{a_{0}k_{ab}^{off}}u^{*}=k_{m}$$

which confirms the dependence of equilibrium bleb size $\left(u^{*}\right)$ on initial intracellular pressure, $F_{0}$, cortex stiffness $k_{c}$ and membrane stiffness $k_{m}$, observed from our model simulations. Additionally, the condition (Eq. 13) points to a dependence of equilibrium bleb size on actin reformation dynamics through the ratio of actin polymerization and depolymerization rate $\frac{k_{abf}^{on}}{k_{ab}^{off}}$. However, the degradation rate of the actin scar (old cortex), cytoplasmic viscosity and cortex permeability appear to have no effect on the equilibrium bleb size. Details of the autonomous reformulation of the model and derivation of the condition Eq. 13 for equilibrium bleb size can be found in the supplemental methods.

**Fig. 5:**
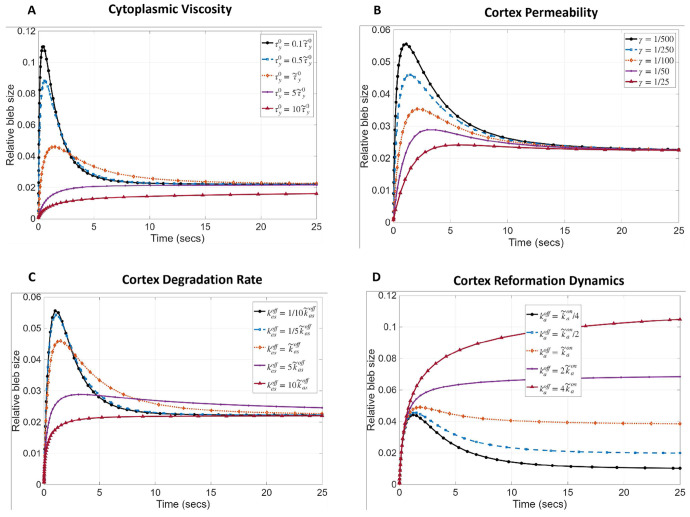
Influence of actin reformation dynamics on bleb size. Effect of A) cytoplasmic viscosity B) cortex permeability, modeled as the fraction of actin concentration in the cortex (actin scar) at the onset of blebbing C) degradation rate of actin scar (old cortex), D) ratio of actin polymerization to depolymerization rate at the reforming cortex (bleb cortex), on bleb size. Bleb size is measured as the strain of the cell boundary at a single point. $\tilde{r}$ is the baseline value of parameter r reported in Table 2.

Finally, we wanted to understand how cortex reformation affects the final bleb size, since this is the primary purpose of developing the mathematical model. We also explore the effect, if any, that cortex degradation, cytoplasmic viscosity and cortex permeability may have on bleb expansion dynamics. Our results, shown in Fig. 5, suggest that a more viscous cytosol will cause blebs to expand monotonically to their resting size, without retracting (Fig. 5A). As the viscosity of the fluid reduces, blebs have a greater initial expansion before retracting sooner to their equilibrium size. Cortex permeability and degradation rate of the actin scar have a similar effect on bleb expansion dynamics (Fig. 5B,C). As predicted theoretically, none of these factors alter the equilibrium size of blebs. In support of our equilibrium analysis, we see that varying the cortex reformation rate changes the equilibrium bleb size (Fig. 5D), with larger blebs forming with a slower cortex reformation rate (or faster depolymerization rate). Interestingly, partial bleb retraction to equilibrium size seems to be regulated by the relative rate of actin polymerization $\left(k_{ab}^{on}\right)$ and depolymerization $\left(k_{ab}^{off}\right)$ at the bleb cortex. Particularly, when $k_{ab}^{off}<k_{ab}^{on}$ no retraction occurs and the blebs expand monotonically to an equilibrium size. On the contrary, blebs retract before reaching equilibrium size when $k_{ab}^{off}\geq k_{ab}^{on}$. Our theoretical model therefore supports the hypothesis that disregulation of polymerization and depolymerization rate of actin during cortex reformation can significantly alter bleb size. In particular, blebs tend to have a larger relative size when the actin depolymerization rate exceeds the polymerization rate.

## 4 Discussion

Paxillin is a scaffolding protein found in focal adhesions that that links the cell membrane to the actin cytoskeleton, and acts as a signaling hub that coordinates adhesion, cell shape, and movement by recruiting proteins that control actin dynamics. Much is known about paxillin’s role in adhesion, cytoskeletal reorganization, and actin-based motility[19, 21, 38, 40-42]. However, Paxillin’s role in bleb-based motility is still poorly understood. We used *D. discoideum* as our model organism as it has a Paxillin orthologue—PaxB— that has highly conserved domains with human Paxillin and maintains much of its mammalian counterpart’s actin-based functions such as being found in focal adhesions and regulating cell-substrate/cell-cell adhesion and motility [19, 43].

We used an under-agarose assay to measure bleb frequency and relative bleb size for both wild-type and *paxB*^−^ blebbing cells under low and high compression. In summary, we found that wild-type blebbing cells respond to a higher compression by increasing bleb frequency and maintaining relative bleb size. However, while *paxB*^−^ respond to increased compression by increasing bleb frequency, as seen in wildtype cells, they increased their relative bleb size, contrary to what was observed in wild type cells. The increase in bleb frequency could be, in part, a response to the increased hydrostatic pressure induced by increased compression, as it is well-documented that cellular hydrostatic pressure drives blebbing[10, 14]. Since loss of PaxB had no impact on the increase in bleb frequency, PaxB does not appear to play a role in this response.

In order to gain insight into the role of PaxB in regulating relative bleb size in response to compression, we developed a one-dimensional mathematical model that describes bleb expansion. Since PaxB is known to regulate actin remodeling, we included a component to account for actin dynamics during the reformation of a new cortex under the expanding bleb. We validated our model by demonstrating that it replicates the known behavior of expanding blebs in response to intracellular pressure, membrane stiffness, cortex stiffness, cytoplasmic and cortex viscosity as well as cortex permeability[25]. Interestingly, when we looked at actin dynamics, our model predicts that the final bleb size increases, much like what we observed in cells lacking PaxB, when the actin reformation rate beneath the forming bleb decreases. However, the cortex degradation rate at the actin scar (old cortex) does not influence the bleb size. Thus, the model points to a possible role of PaxB specifically in regulating the rate of cortex reformation in the growing bleb. This is in keeping with the fact that paxillin is known to enrich the cortex by recruiting a variety of proteins to assemble, stabilize, and bundle actin at adhesion sites[38, 41, 42]. Our model and experimental data suggest that PaxB may be performing a similar role in cortex reformation during bleb stabilization in chemotaxing cells.

It is known that the loss of PaxB causes altered motility for cells migrating under agarose towards cAMP [39]. Our model and data suggest that this altered motility may be due to improper regulation of bleb-based motility. Our data show that PaxB does in fact contribute to the regulation of motility under compression, in particularly that PaxB is needed to regulate the relative size of blebs. Through a combination of experimentation and mathematical modelling, we have identified a novel role and potential mechanism for paxillin in regulating bleb-based motility. This role and mechanism may help explain how disruptions in paxillin regulation cause a variety of developmental disorders and cancers, including metastatic cancers[41, 42, 44], and why, paxillin null fibroblast cells have increased invasion and migration in a 3-D Matrigel[21]. Based on the success of this work, we plan to expand the one-dimensional model developed here into a comprehensive two-dimensional model of bleb-based motility. By paring the advanced model with experimentation as we have done here, we should be able to identify other proteins and ascertain their potential mechanisms of action in bleb-based chemotaxis.

## Supplementary Material

This is a list of supplementary files associated with this preprint. Click to download.

• PaxillinPapersupplementaryinfo.pdf

## Figures and Tables

**Table 1:** Summary of experimental data. This table summarizes the data analyzed. The “Total cells analyzed” reflects counts for all cells that produced at least one bleb and were not in contact with other cells.

	Ax-2 0.4%	*paxB*^−^ 0.4%	Ax-2 0.7%	*paxB*^−^ 0.7%
Total cells analyzed	30	17	20	32
Total number of blebs	133	71	165	252

**Table 2:** Parameter values for simulating the bleb expansion models

Parameter	Description	Value	References
$F_{0}$	Resting Intracellular pressure	$0.04nN/(\mu m{)}^{2}$	[36]
m	Rate at which driving pressure decreases in 1D model	0.1	This work
$\tau_{c}^{0}$	Viscosity coefficient mature cortex	$0.064nNs/(\mu m{)}^{3}$	[32]
$a_{0}$	Actin density in mature cortex	1.8	[31]
$k_{ab}^{on}$	Actin polymerization rate at bleb boundary	0.7602	[31]
$k_{ab}^{off}$	Actin depolymerization rate at bleb boundary	0.4344	[31]
$k_{as}^{off}$	Actin depolymerization rate at actin scar	0.0120	[31]
$\tau_{y}^{0}$	Viscosity coefficient of cytoplasm	$6.09nNs/(\mu m{)}^{3}$	[32]
$k_{c}$	Spring constant for the cortex	$0.098nN/(\mu m{)}^{3}$	[32]
$k_{m}$	Spring constant for membrane	$0.00392nN/(\mu m{)}^{3}$	Estimate using [32]
θ	Ratio of membrane stiffness to cortical stiffness	1/250	[25]
$k_{a}$	Strength of single linker protein	$10^{-1}nN/(\mu m)$	[28]
$\rho_{0}$	Total available density of linker protein	$10^{2}(\mu m{)}^{-2}$	[28]
$k_{\text{on }}$	Binding rate of linker protein	$10^{4}(s{)}^{-1}$	[28]
$k_{off}^{0}$	Detachment rate of linker proteins at zero displacement	$10(s{)}^{-1}$	[28]
β	1/Thermal energy ${\left(k_{B}T\right)}^{-1},k_{B}$ is Boltzmann constant and T is thermodynamics temperature	$\frac{10^{6}}{4.1}((nN)(um){)}^{-1}$	[28]
δ	characteristic bond length	$10^{-3}(\mu m)$	[28]
